# Supplementary material for: High magnesium mobility in ternary spinel chalcogenides
Source: Nat Commun. 2017 Nov 24;8:1759. doi: 10.1038/s41467-017-01772-1 (PMC5700915; doi:10.1038/s41467-017-01772-1)
Supplement: Supplementary file 1 — Supplementary Information [file 41467_2017_1772_MOESM1_ESM.pdf]

SUPPLEMENTARY FIGURES

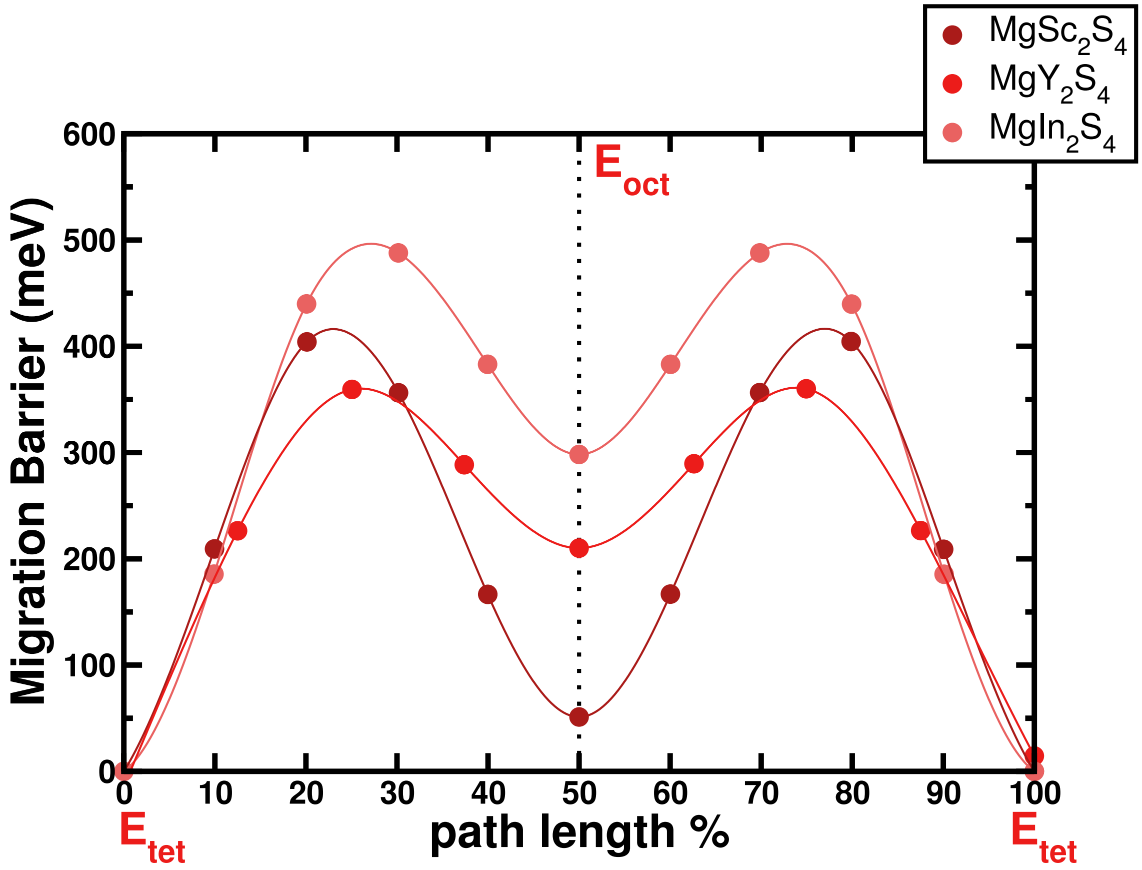

Supplementary Figure 1 First-principles Nudged Elastic Band results for Mg^{2+} migration energies (in meV) in MgX_2S_4 frameworks. Relevant energy sites $\boldsymbol{E_{tet}}$ and $\boldsymbol{E_{oct}}$ are indicated (see **Figure 1a** and **Figure 1b** in the main text).

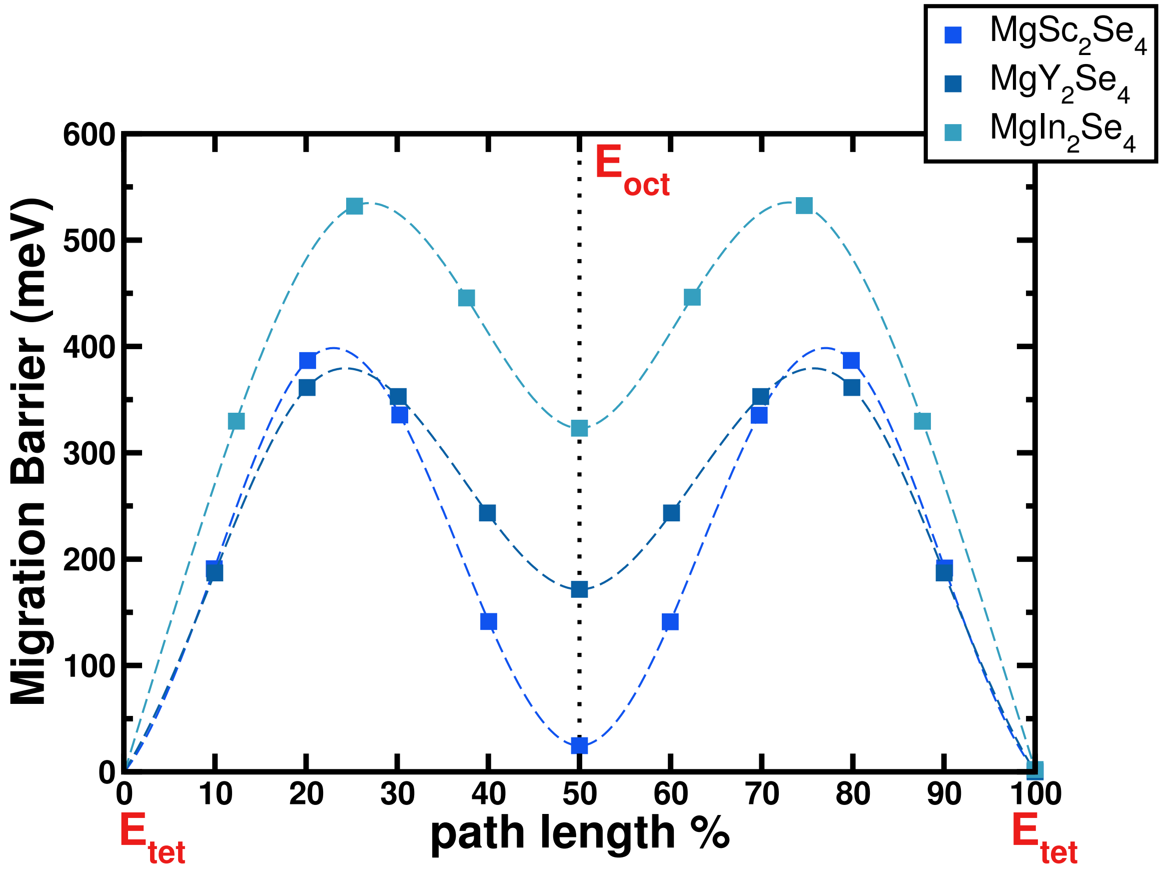

Supplementary Figure 2 First-principles Nudged Elastic Band results for Mg^{2+} migration energies (in meV) in MgX_2Se_4 frameworks. Relevant energy sites $\boldsymbol{E_{tet}}$ and $\boldsymbol{E_{oct}}$ are indicated (see **Figure 1a** and **Figure 1b** in the main text).

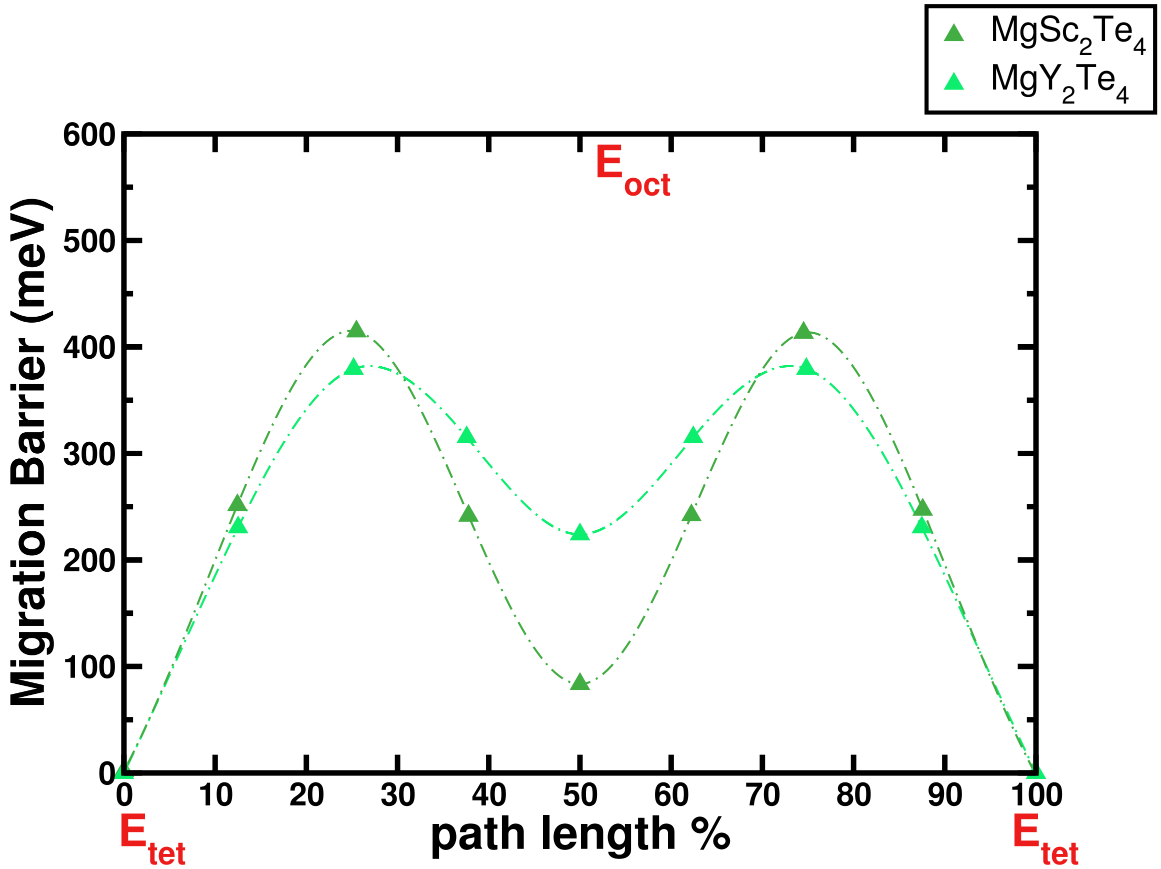

Supplementary Figure 3 First-principles Nudged Elastic Band results for Mg^{2+} migration energies (in meV) in MgX_2Se_4 frameworks. Relevant energy sites $\boldsymbol{E_{tet}}$ and $\boldsymbol{E_{oct}}$ are indicated (see **Figure 1a** and **Figure 1b** in the main text).

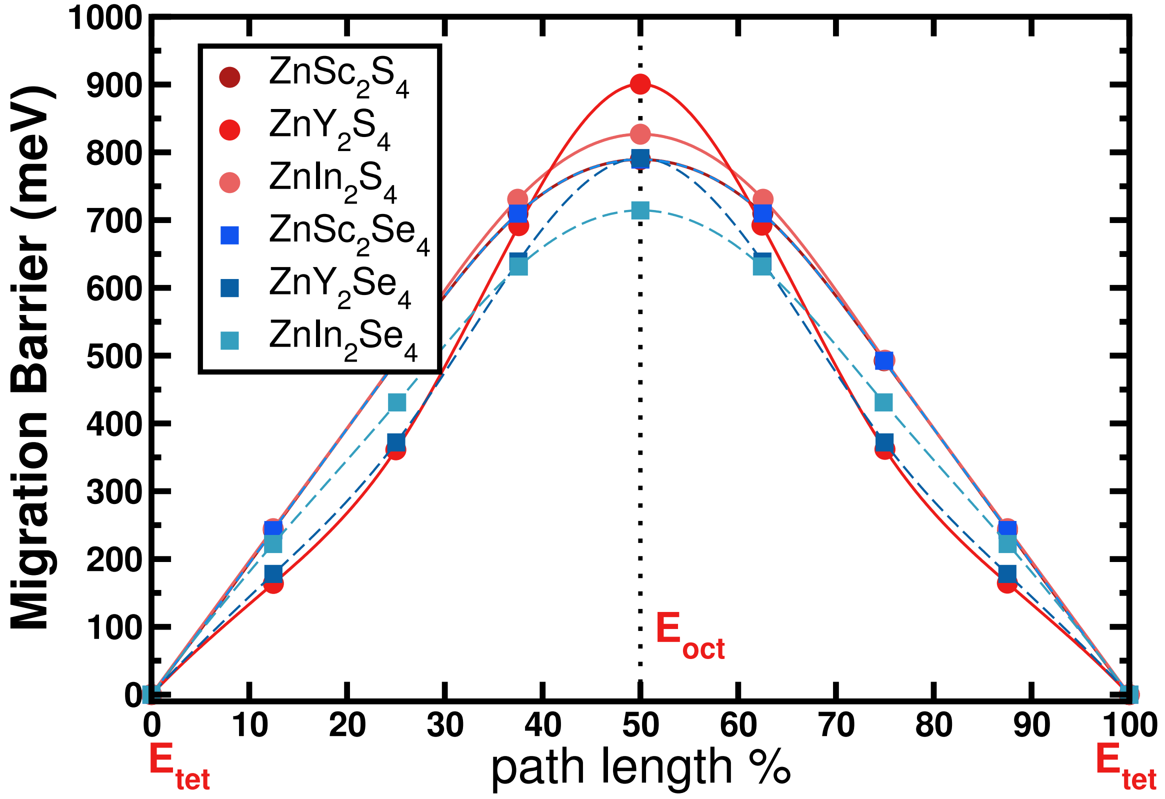

Supplementary Figure 4 First-principles Nudged Elastic Band results for Zn^{2+} migration energies (in meV) in ZnX_2Z_4 frameworks. Relevant energy sites $\boldsymbol{E_{tet}}$ and $\boldsymbol{E_{oct}}$ are indicated.

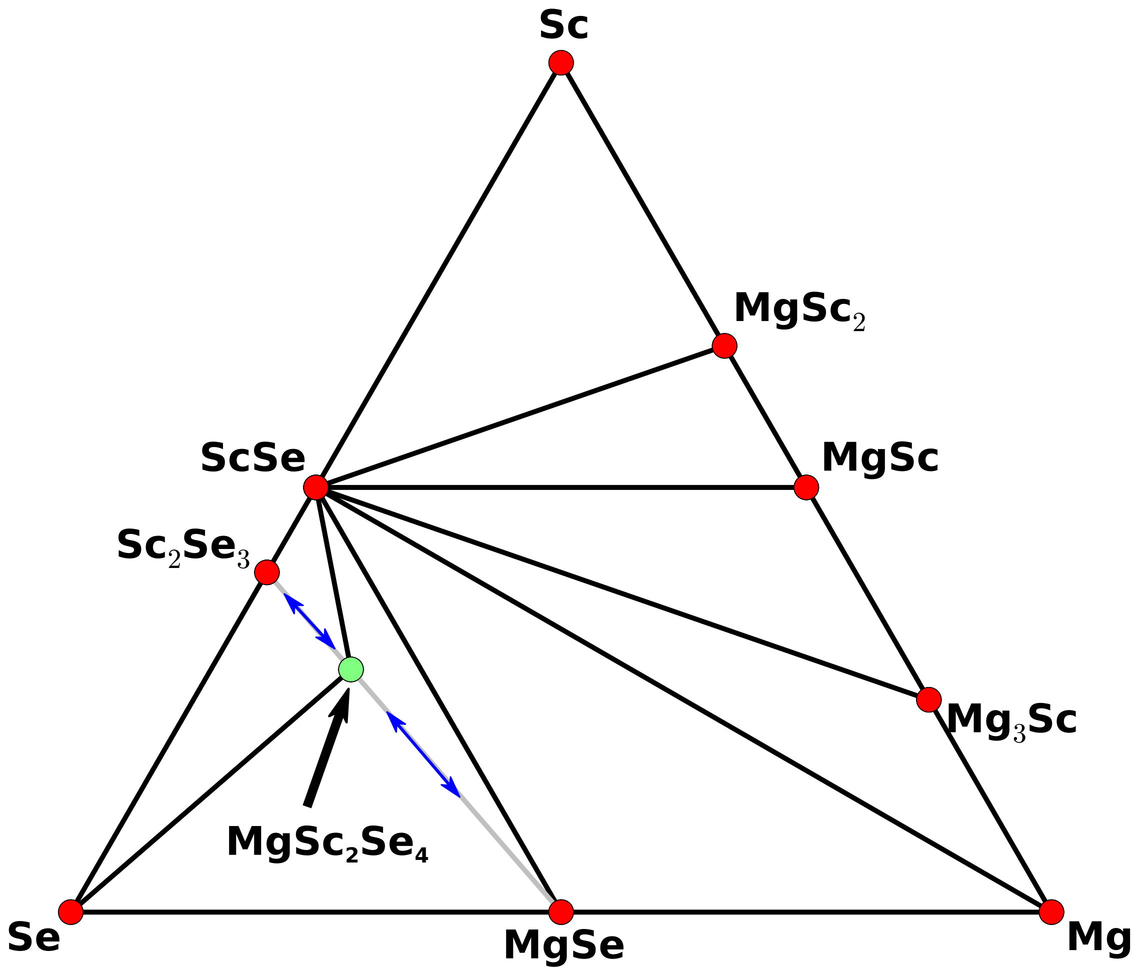

Supplementary Figure 5 Ternary phase diagram for $MgSc_2Se_4$ with tie lines (in grey and blue arrows) connecting the binary compounds MgSe and Sc_2Se_3 to the desired ternary phase.

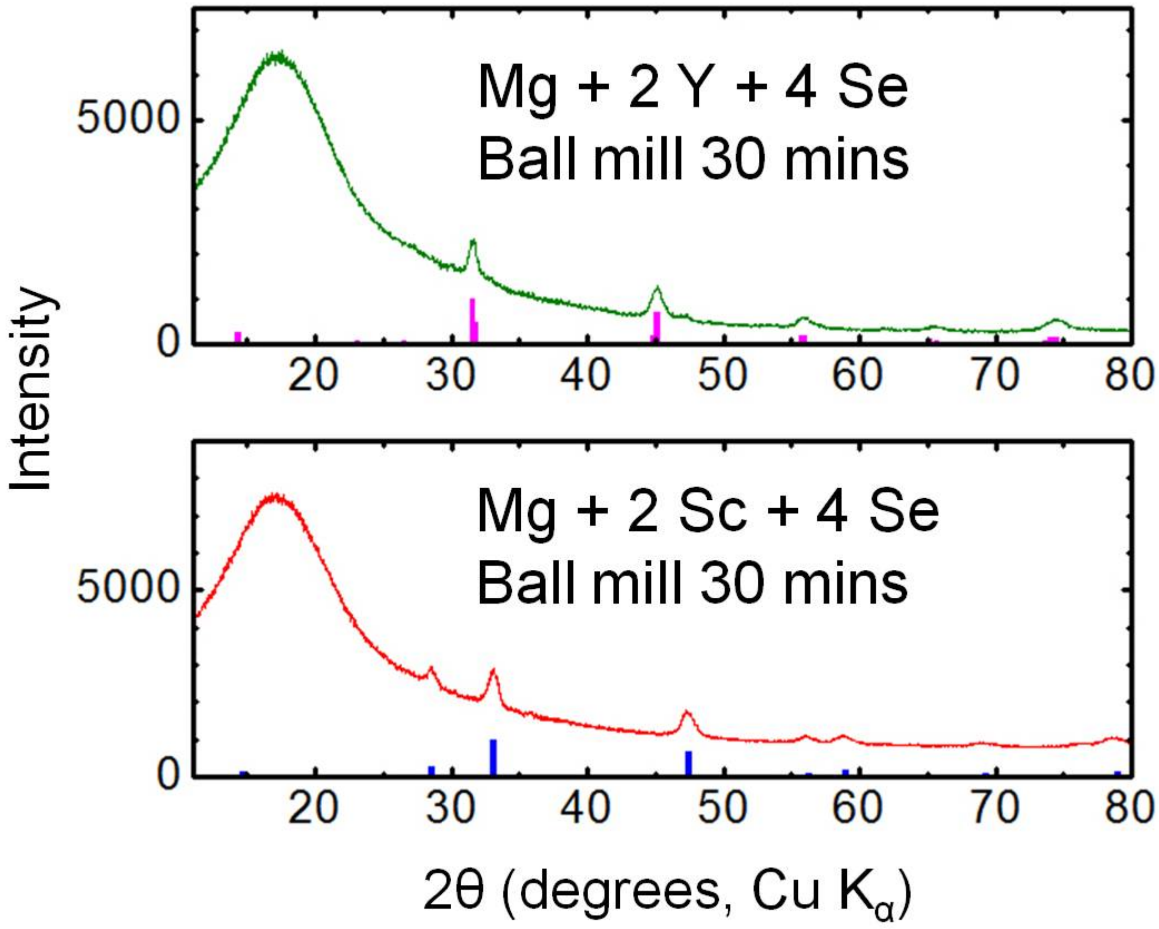

Supplementary Figure 6 X-ray diffraction patterns of the Mg, Y(Sc), and Se precursors which were ball milled in a tungsten carbide jar for 30 minutes. The broad feature observed in the XRD patterns below approximately 25 degrees (2θ) is due to the contribution from the kapton tube which was used to secure the power sample without any air exposure.

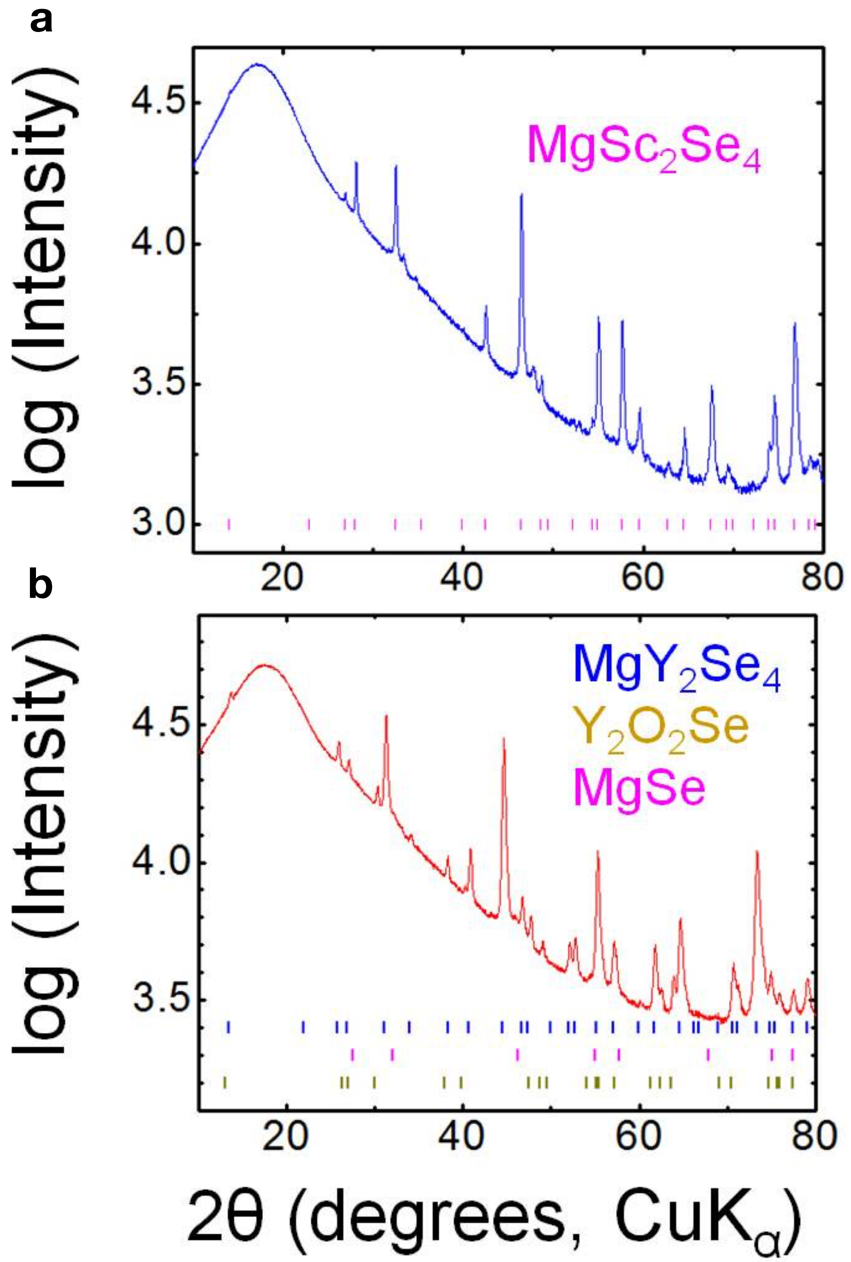

Supplementary Figure 7 X-ray diffraction patterns of the as-prepared $MgSc_2Se_4$ (**a**) and MgY_2Se_4 (**b**) samples. For clarity, the log of intensity is displayed on the y-axis.

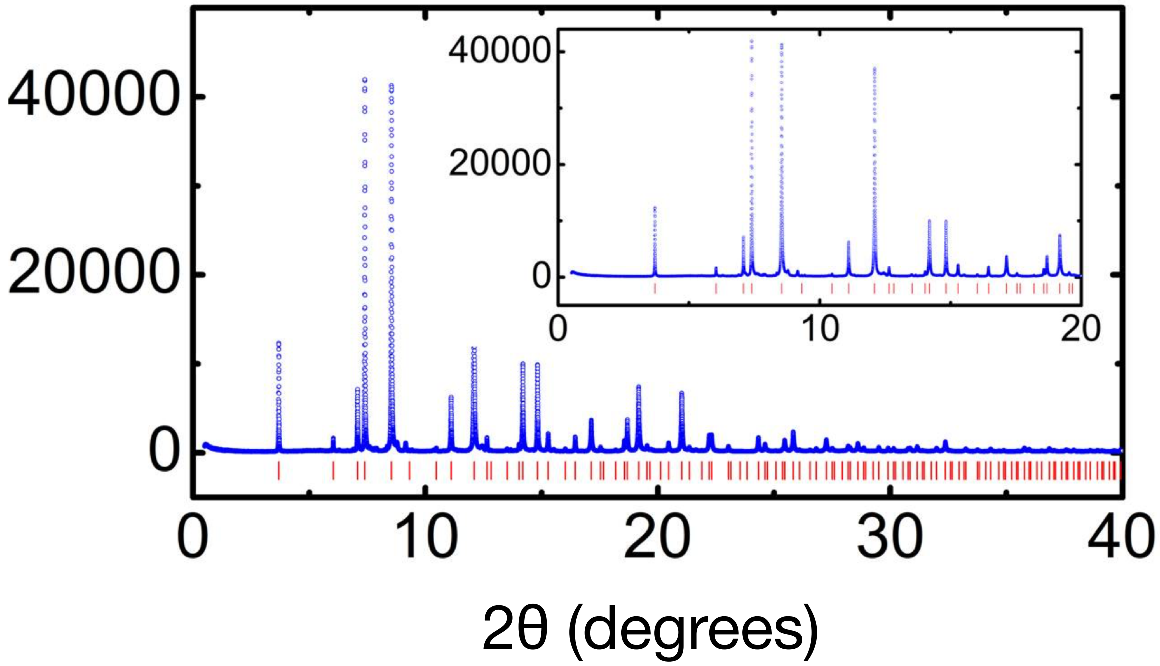

Supplementary Figure 8 Synchrotron X-ray diffraction pattern of the $MgSc_2Se_4$ sample after a second heat at 1000 °C for 100 hours. The diffraction peaks corresponding to spinel $MgSc_2Se_4$ are shown with red tick marks. The data was collected at beamline 11 BM of the Advanced Photon Source, Argonne National Laboratory, with a wavelength of 0.414173 Å.

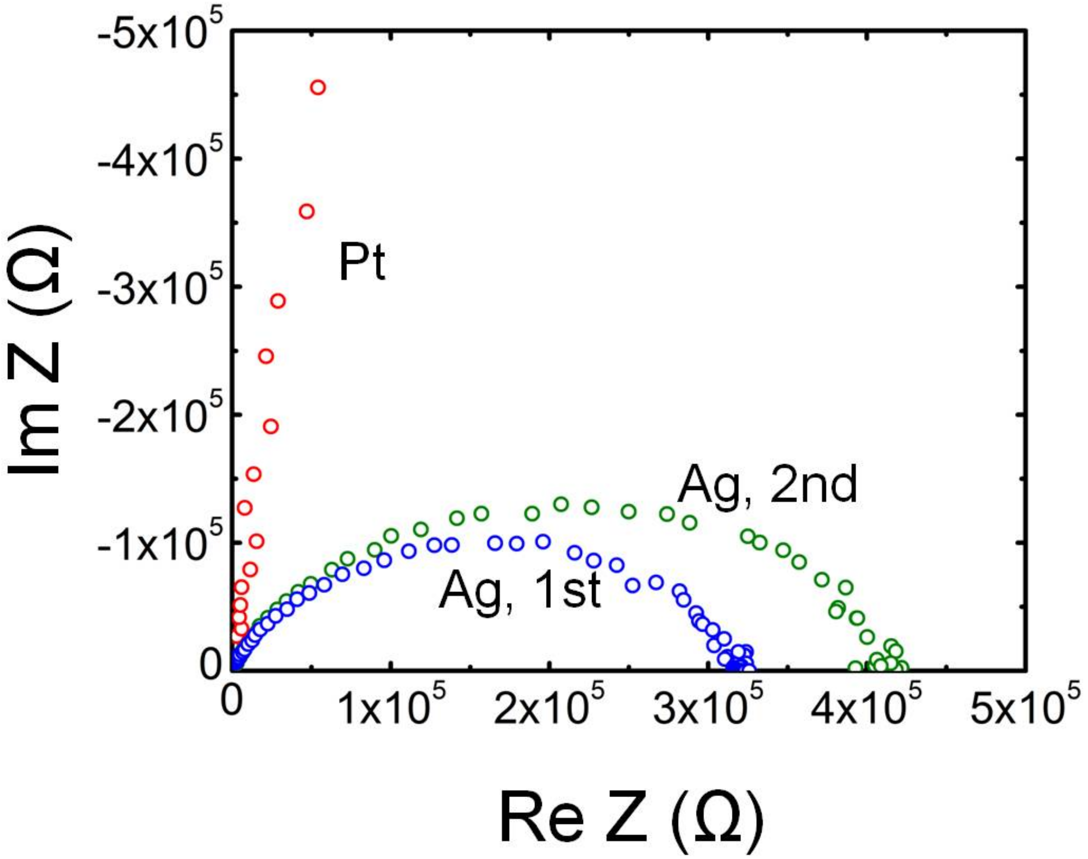

Supplementary Figure 9 Impedance spectra of $MgSc_2Se_4$ pellets of similar-thickness (0.78 mm and 0.86 mm for Ag and Pt, respectively) with Ag or Pt as blocking electrodes. The Ag blocking electrode was formed through applying and drying of Ag paste (SPI) on both sides of the pellet. The Pt blocking electrode was formed through sputter deposition. The impedance of the Ag/$MgSc_2Se_4$/Ag cell increases from approximately 300 to 400 kΩ, when the impedance measurement was performed after 40 minutes. "Ag, 1st" was used to denote the impedance data (blue circles) right after the assembly of Swagelok cell, and "Ag, 2nd" was used to denote the data (olive circles) which was collected after 40 minutes. Both measurements were performed under Ar protection. The impedance data with deposited Pt as blocking electrodes was displayed in red circles.

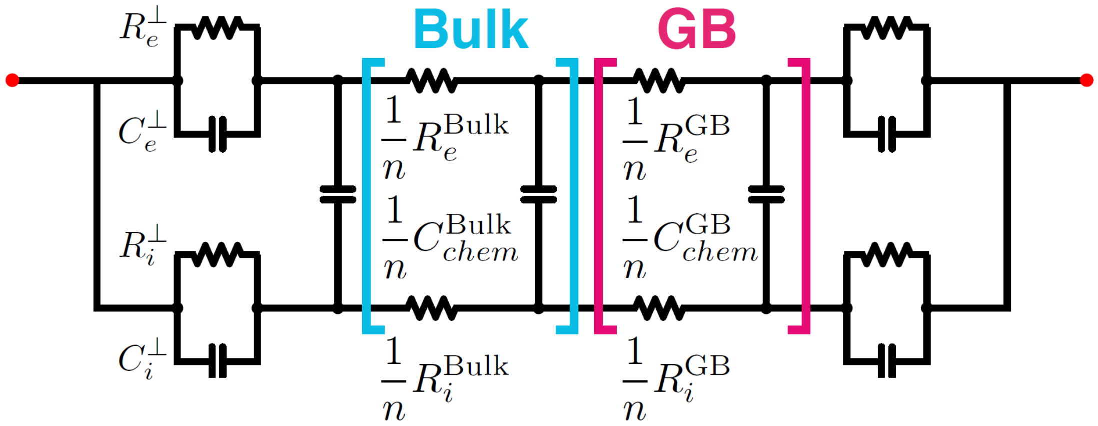

Supplementary Figure 10 The circuit model used to fit the impedance data of Ta/$MgSc_2Se_4$/Ta, which includes two Jamnik-Maier elements.[1–3] These two elements were tentatively attributed to contributions from bulk and grain boundary.

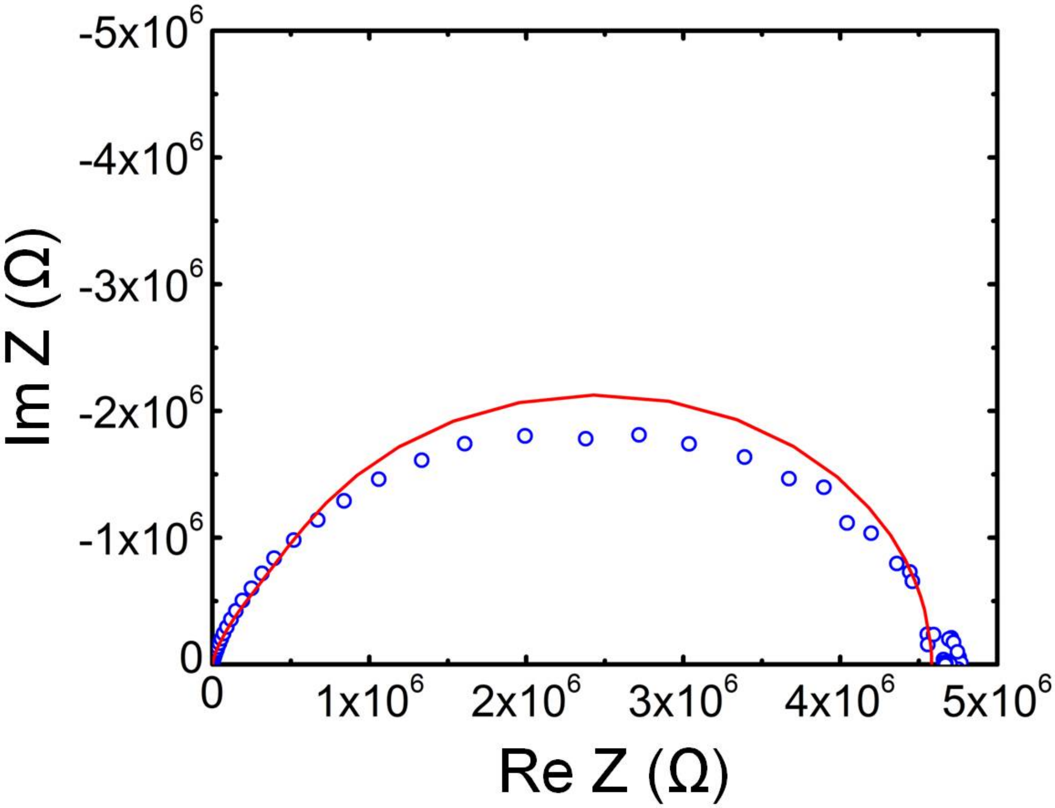

Supplementary Figure 11 The fitting (red line) of the impedance data for Ta/$MgSc_2Se_4$/Ta (blue circles) with the use of only one Jamnik-Maier element.[2,3]

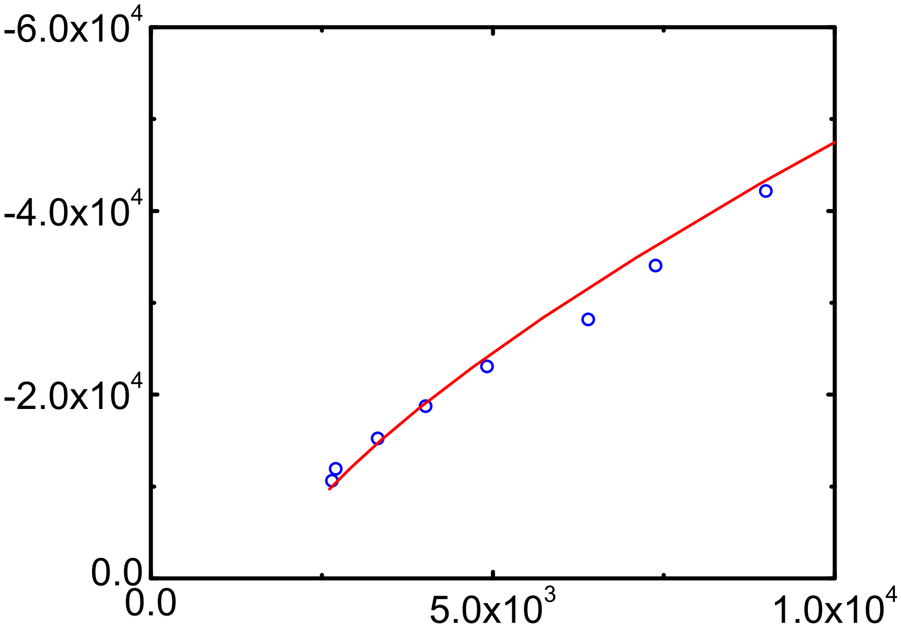

Supplementary Figure 12 Close-up impedance data of **Supplementary Figure 11** (blue circles) at low R_e Z values.

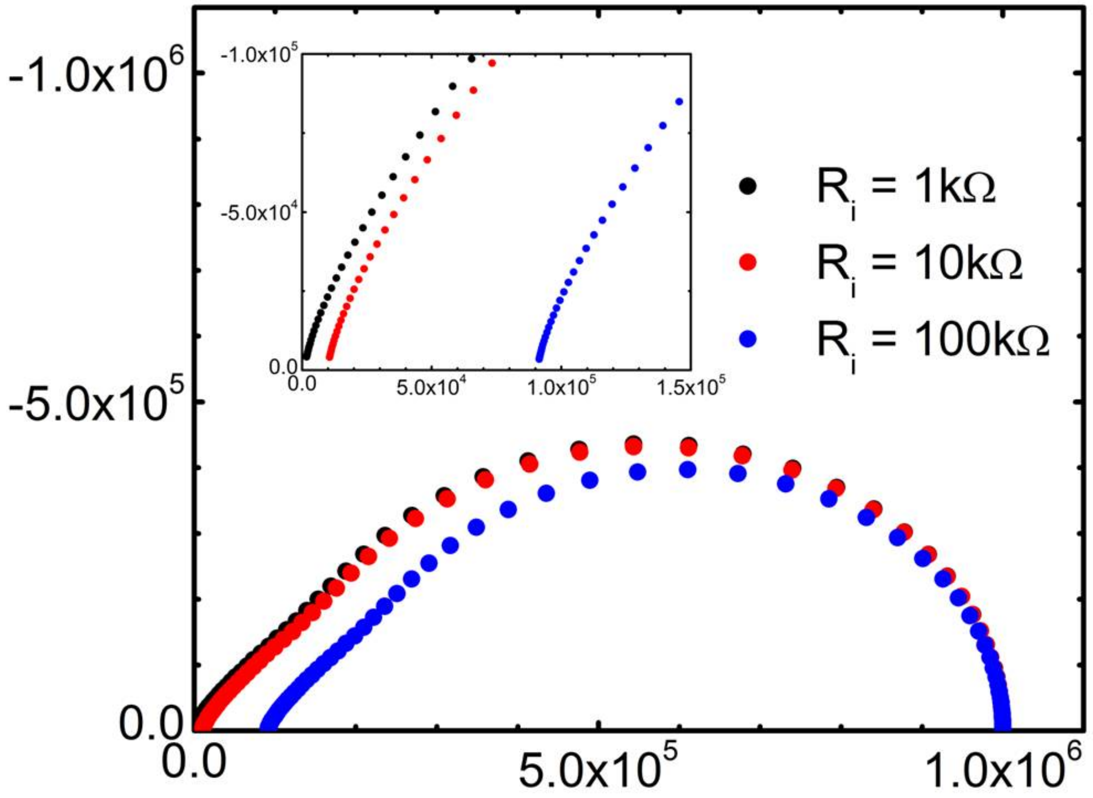

Supplementary Figure 13 Simulated impedance spectra based on the Jamnik-Maier model (from 1 MHz to 0.1 Hz).[1] The ionic resistance (R_i) is varied from 1 kΩ to 100 kΩ, with the electronic resistance fixed to 1 MΩ. All other parameters are kept constant for the three simulated spectra. The inset shows a close-up of the high frequency portion.

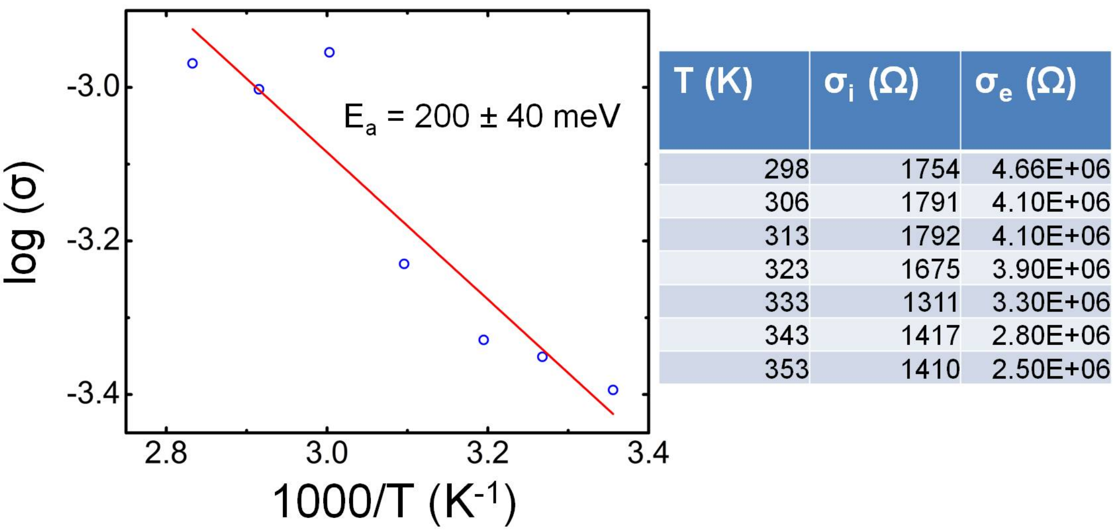

T (K)	σ_i (Ω)	σ_e (Ω)
298	1754	4.66E+06
306	1791	4.10E+06
313	1792	4.10E+06
323	1675	3.90E+06
333	1311	3.30E+06
343	1417	2.80E+06
353	1410	2.50E+06

Supplementary Figure 14 Conductivity (S cm^{-1}) vs. temperature (K) plot of the $MgSc_2Se_4$ sample exhibits an Arrhenius behavior with an activation barrier of ~200 ± 40 meV. The extracted values of ionic and electronic resistance are tabulated on the right.

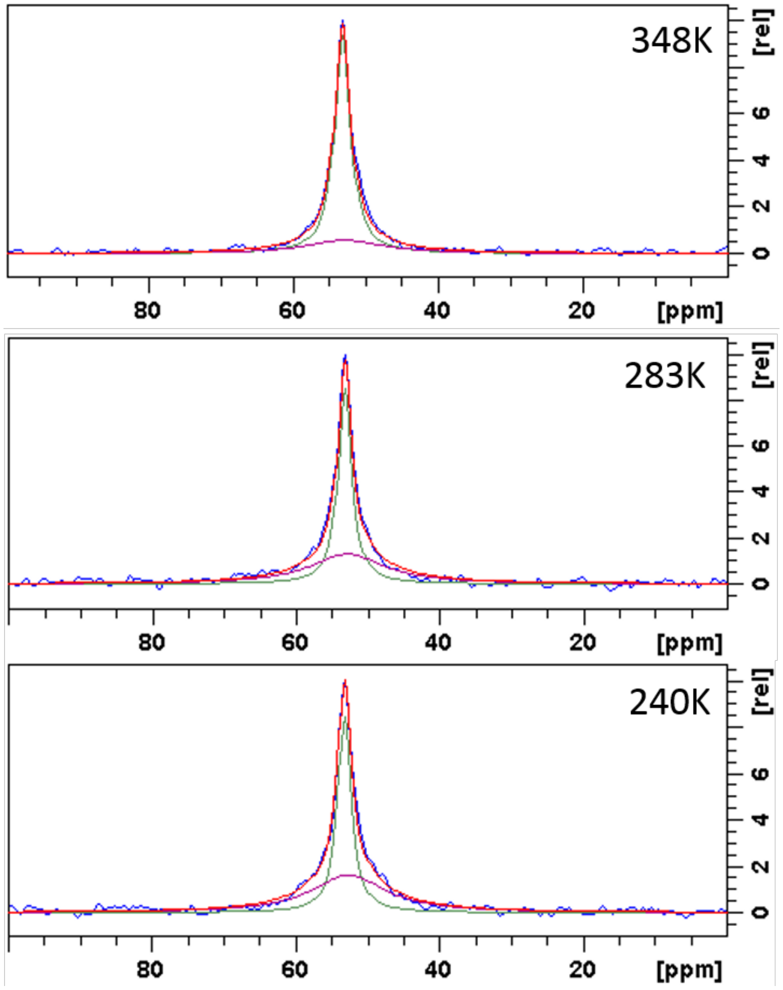

Supplementary Figure 15 Quadrupolar fit of the ^{25}Mg MAS Variable Temperature NMR spectra of $MgSc_2Se_4$ collected at 11.7 T with spinning speed of 20 kHz.

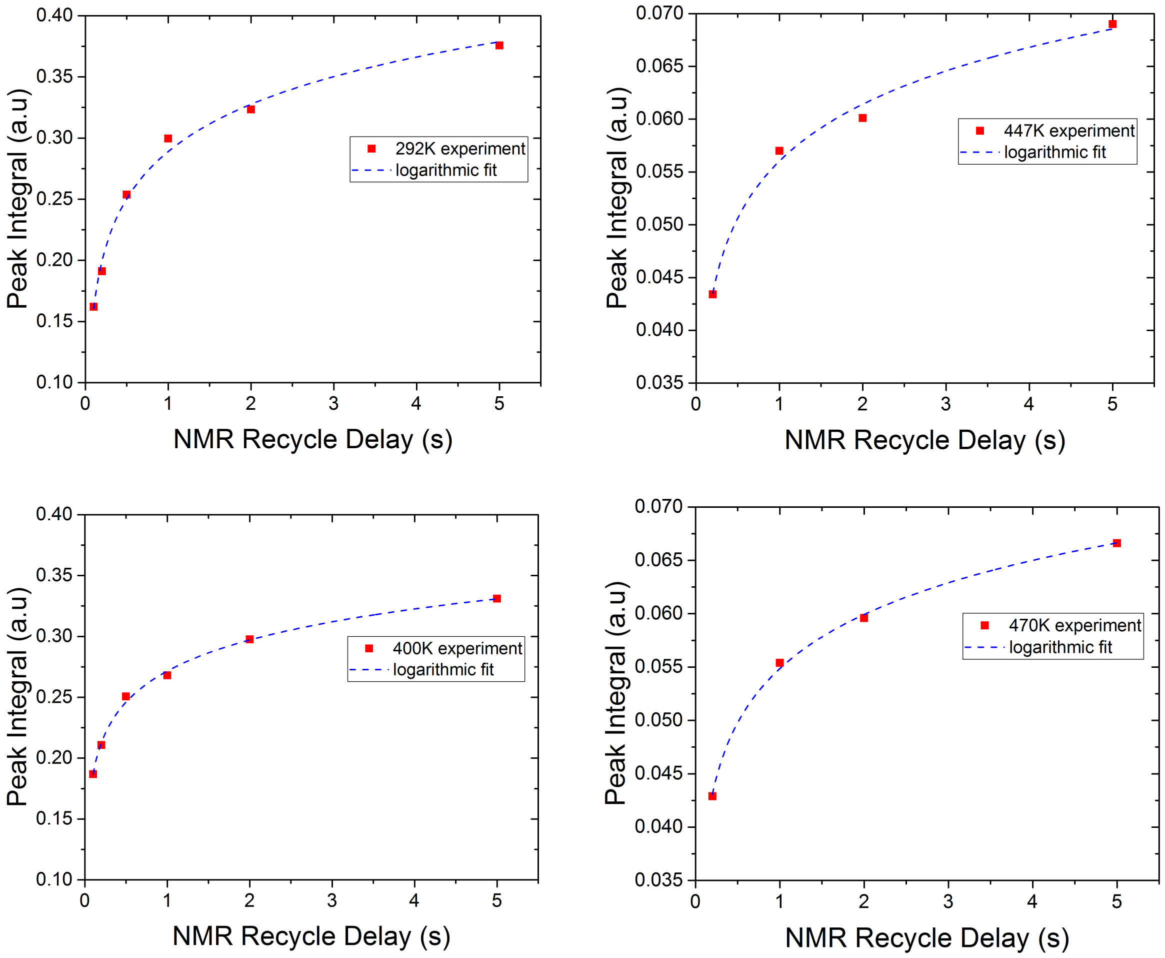

Supplementary Figure 16 Logarithmic fits on static ^{25}Mg NMR signals for $MgSc_2Se_4$ collected at 292 K, 400K, 447K and 470K, respectively using a magnetic field of 7.02 Tesla.

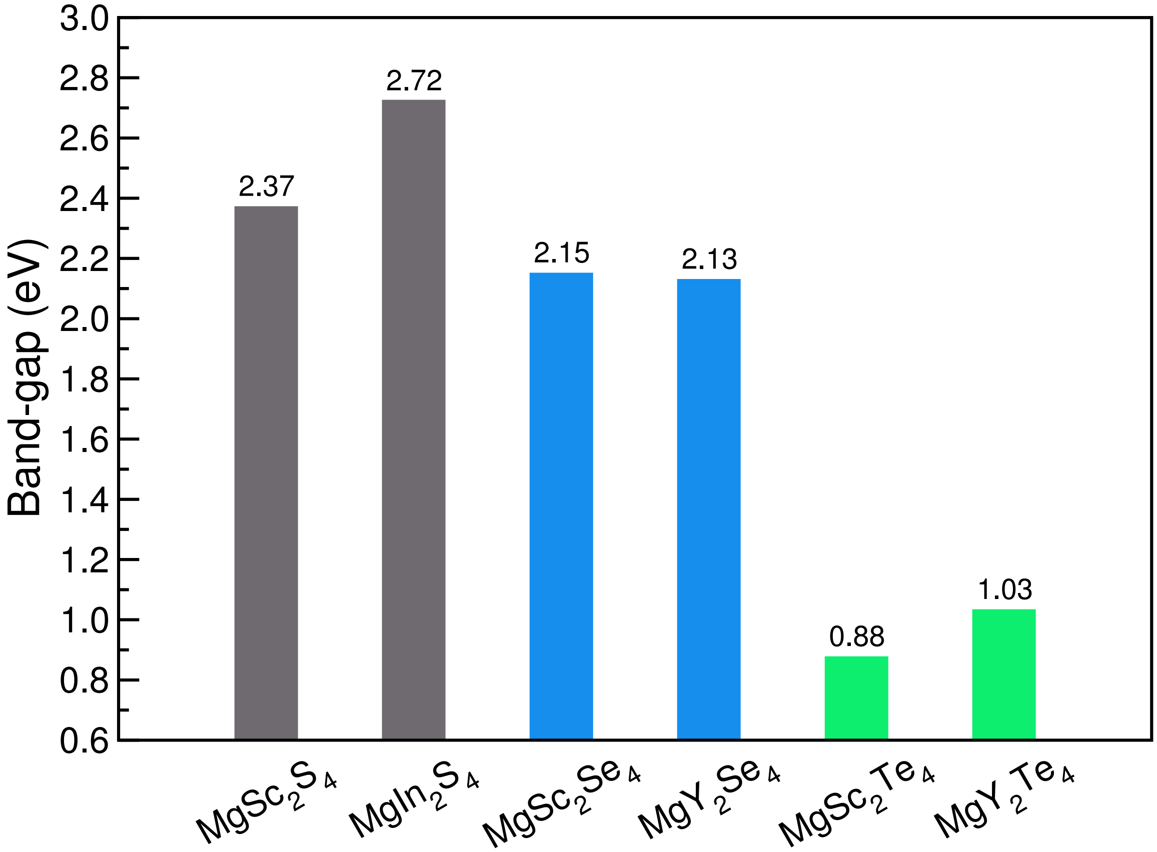

Supplementary Figure 17 Computed intrinsic Band gap for the chalcogenides computed using HSE06.

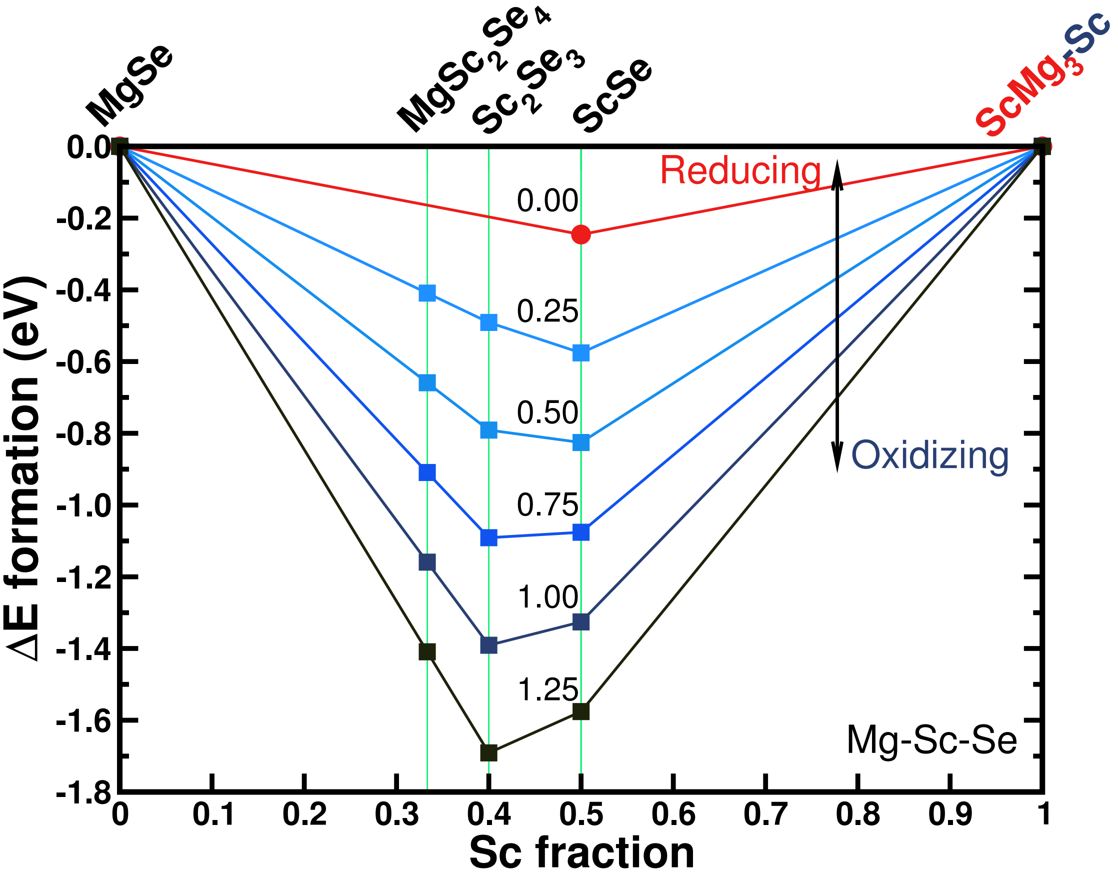

Supplementary Figure 18 Mg-Sc-Se grand-potential phase diagram as function of Sc fraction and various Mg chemical potentials. The red-dots and blue-squares indicated only the stable phases. Green lines identified the fraction of some stable phases. Numbers inside the graph indicate the voltage of each line.

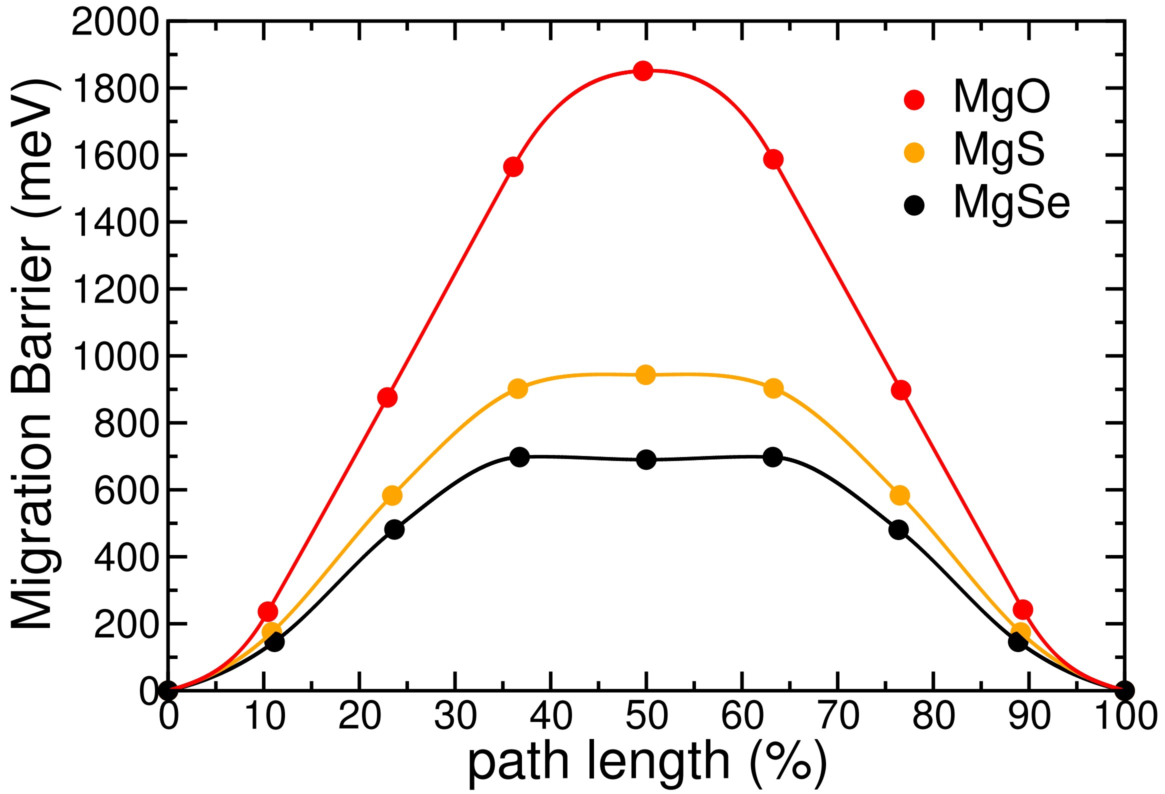

Supplementary Figure 19 First-principles Nudged Elastic Band results for Mg^{2+} migration energies (in meV) in MgZ_2 frameworks, with X= O, S and Se.

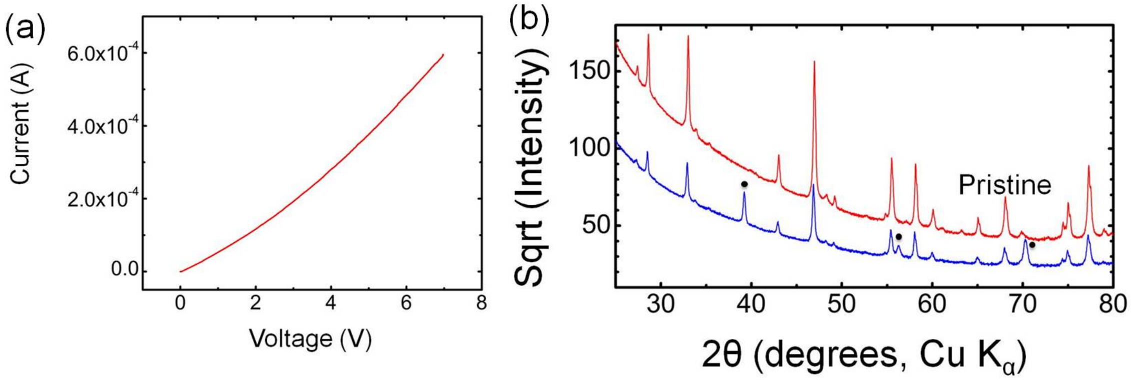

Supplementary Figure 20 Electrochemical and *ex situ* X-ray characterization of a symmetric Ta/$MgSc_2Se_4$/Ta cell. Linear sweep voltammetry (LSV) of the **(a)** Ta/$MgSc_2Se_4$/Ta cell. The X-ray diffraction patterns of the electrolyte before and after the CV measurement are presented in **(b)**, where red curves correspond to the as-prepared $MgSc_2Se_4$ and the blue curves to the sample after LSV scan. The peaks denoted with black dots in **(b)** are associated with Ta metal which are distinct from the electrolyte powder.

SUPPLEMENTARY TABLES

Supplementary Table 1: DFT enthalpy of formation at 0 K of binary and ternary chalcogenides.

Reaction	ΔE	
	kJ mol^{-1}	eV
Mg + Se → MgSe	–242.1	–2.50
Y + Se → YSe	–339.7	–3.52
Sc + Se → ScSe	–306.8	–3.18
2Y + 3Se → Y_2Se_3	–888.8	–9.20
2Sc + 3Se → Sc_2Se_3	–802.6	–8.11
Mg + 2Y + 4Se → MgY_2Se_4	–1152.7	–11.95
Mg + 2Sc + 4Se → $MgSc_2Se_4$	–1062.0	–11.00
MgSe + Y_2Se_3 → MgY_2Se_4	–21.8	–0.23
MgSe + Sc_2Se_3 → $MgSc_2Se_4$	–10.7	–0.11
MgSe + 2YSe + Se → MgY_2Se_4	–231.3	–2.40
MgSe + 2ScSe + Se → $MgSc_2Se_4$	–785.1	–8.13

Supplementary Table 2 Crystallographic data for $MgSc_2Se_4$ based on the Rietveld refinement of the synchrotron X-ray data.

Radiation	Synchrotron X-ray (11BM, APS)
Crystal system	Cubic
Space group	*Fd-3m* (#227)
Lattice parameters	$a = b = c$ = 11.11823(3) Å
Cell volume	1374.38(1) $Å^3$
Density (calculated)	4.15654 g cm^{-3}
λ	0.414173 Å
R_{wp}	10.082%
R_p	8.018%
χ^2	1.522

Supplementary Table 3 Atomic site information for $MgSc_2Se_4$.

Atom	Wyck.	*x/a*	*y/b*	*z/c*	Occ.	B_{iso} ($Å^2$)
Mg	8b	3/8	3/8	3/8	1	1.45(4)
Sc	16c	0	0	0	1	0.70(1)
Se	32e	0.24259(1)	0.24259(1)	0.24259(1)	1	0.75(1)

Supplementary Table 4 Selected bond distances (in Å) for $MgSc_2Se_4$.

Mg–Se (x4)	2.5499(3)
Sc–Se (x6)	2.6997(1)

Supplementary Table 5 Example of a Materials Project database query to identify sulfides Mg conductors. 'icsd_ids.0': {'$exists': True} indicates that the search was carried out only on materials available in the Inorganic Chemical Structure Database.

```
{'elements': {'$all': ['Mg', 'S'],
          '$nin': ['Ti', 'V', 'Cr', 'Mn', 'Fe', 'Co', 'Ni',
'Cu', 'Nb', 'Mo', 'Tc', 'Ru', 'Rh', 'Pd', 'Ag', 'Ta', 'W',
'Re', 'Os', 'Ir', 'Pt', 'Au', 'H', 'C', 'Li', 'Na', 'K',
'O']},
              'icsd_ids.0': {'$exists': True},
}
```

Supplementary Table 6 ICSD sulfide materials identified according to the criteria exposed above sorted by Volume per anion (in Å^3). Mg coordination in its stable site is also reported. MP id identifies the compounds in the Materials Project.

MP id	ICSD id	Formula	Red. Formula	Volume/Anion	Mg coord.
mp-3872	38344	$Mg_4Al_8S_{16}$	$MgAl_2S_4$	34.21	6
mp-1315	41234	MgS	MgS	35.74	6
mp-17441	23525	$Mg_8Ge_4S_{16}$	Mg_2GeS_4	36.84	6
mp-667323	15350	$Mg_6Ga_{12}S_{24}$	$MgGa_2S_4$	37.64	6
mp-14307	37423	$Mg_2Sc_4S_8$	$MgSc_2S_4$	38.30	4
mp-866517	606475	$Mg_2Ce_6Al_2S_{14}$	$MgCe_3AlS_7$	38.60	6
mp-20493	53096	$Mg_2In_4S_8$	$MgIn_2S_4$	40.43	4
mp-14304	37420	$Mg_2Lu_4S_8$	$MgLu_2S_4$	41.53	4
mp-8272	37417	$Mg_2Yb_4S_8$	$MgYb_2S_4$	44.17	4

Supplementary Table 7 ICSD selenide materials identified according to the criteria exposed above sorted by Volume per anion (in Å^3). Mg coordination in its stable site is also reported. MP id identifies the compounds in the Materials Project.

MP id	ICSD id	Formula	Red. Formula	Volume/Anion	Mg coord.
mp-10760	53946	MgSe	MgSe	41.84	6
mp-29624	41928	$Mg_2Al_2Se_5$	$Mg_2Al_2Se_5$	43.86	6
mp-1001019	642814	$Mg_2Sc_4Se_8$	$MgSc_2Se_4$	44.20	4
mp-30943	413165	$Mg_2P_2Se_6$	$MgPSe_3$	45.01	6
mp-9479	83363	$MgAl_2Se_4$	$MgAl_2Se_4$	45.71	6
mp-10191	44912	$Mg_2Lu_4Se_8$	$MgLu_2Se_4$	47.82	4
mp-15802	76051	$Mg_2Tm_4Se_8$	$MgTm_2Se_4$	48.57	4
mp-15803	76052	$Mg_2Y_4Se_8$	MgY_2Se_4	50.10	4
mp-15804	76053	$Mg_2Yb_4Se_8$	$MgYb_2Se_4$	50.76	4

SUPPLEMENTARY NOTES

Supplementary Note 1: Mg and Zn migration barrier and topology in AX_2Z_4

The corresponding energy profile for the *tet-oct-tet* topology, of **Supplementary Figures 1**, **2** and **3**, typically possesses a "valley" shape, with the *oct* site at a higher energy (E_{oct}) than the *tet* site (E_{tet}), while the difference in energy between the *oct* and *tet* sites can be influenced by the metal ion (X in MgX_2Z_4). The migration energy profiles of Zn in **Supplementary Figure 4** display a "plateau" behavior with the barrier maxima corresponding to the *oct* site (instead of the shared triangular face), and in agreement with previous results.[4,5]

Supplementary Note 2: Synthesis and structural characterization of selenide spinels

The computed (GGA) formation enthalpies of $MgSc_2Se_4$ and MgY_2Se_4 are extremely negative as shown in **Supplementary Table 1**. The reaction enthalpies to form the ternary spinel from the binary mixtures, in contrast, are negative but much smaller in magnitude. These results suggest that the ternary spinel can be synthesized through mixing and reacting the elements. Hence, the preliminary synthesis of binary precursors is unnecessary.

The synthesis of the selenide compound was found to be extremely challenging, with difficulties associated to the air sensitivity of selenide which can easily react with constituents in the air or residual oxygen in the precursors, producing oxygen containing impurities. Also, the relatively low melting and boiling points of the selected precursors (such as Mg metal, *mp*:650 °C, *bp*:1091 °C) can drive the system into off-stoichiometry, due to the high volatility of the precursors at the synthesis temperature (1000 °C). These difficulties were partially overcome through preliminary ball milling and a pelleting process in the Ar glove box. These treatments substantially enhanced the reaction kinetics. As shown in **Supplementary Figure 6**, the binary compounds with high melting points were already formed after ball milling. A subsequent sintering of the ball milled precursors produced the MgY_2Se_4 and $MgSc_2Se_4$ spinels (**Supplementary Figure 7**).

Unfortunately, while high purity $MgSc_2Se_4$ can be obtained, the as-prepared MgY_2Se_4 samples always contain Y_2O_2Se as a major impurity phase. While Y_2O_2Se is a

thermodynamically stable compound, no such equivalent Sc_2O_2Se compound exists, which explains the different behavior in the syntheses of $MgSc_2Se_4$ and MgY_2Se_4. Two possible sources of oxygen causing the production of O-containing impurity phases can be envisioned: *i*) the presence of small amounts of metal oxides in the precursors of Mg or Y, and *ii*) oxygen leaking during the sintering process. Since Y metal is conventionally obtained through the reduction of the corresponding metal oxide, we believe that the former scenario is more likely.

The structure of $MgSc_2Se_4$ was further refined using the Rietveld method. The diffraction pattern of $MgSc_2Se_4$ can be readily indexed with the spinel lattice (*Fd-3m*, a = b = c = 11.11823 Å). When the site occupancies of Mg, Sc and Se were tested individually, close-to-full occupancy was obtained for the Sc and Se sites (~ 0.99), and the Mg site occupancy was calculated to be ~ 0.97. The R_{wp} (figure-of-merit for the fitting quality) with 0.97 Mg occupancy is 10.076, which is only marginally lower compared with that of full Mg occupancy (10.082). These R_{wp} (10.076 vs. 10.082) and occupancy differences (0.97 vs. 1.00) are very well within the error of diffraction analysis. The presence of Mg vacancy, thus, cannot be confirmed based on this data only. Site inversion of Mg, Sc which occurs in many spinel compounds was not detected. The final refinement was therefore carried out utilizing a normal $MgAl_2O_4$-spinel model. Structural parameters from the refinement are tabulated in **Supplementary Tables 2-4**.

Supplementary Note 3: Impedance spectroscopy

Ionic conductivity of solid-state materials can be determined through electrochemical impedance spectroscopy. The characterization of ionic conductivity of a compound often requires the use of two identical "ion blocking" electrodes on both sides of the pellet of the solid conductors. An ideal ion blocking electrode should completely block the interface transfer of ions between the solid conductor and the electrodes, and is completely permeable to electrons. The blocking electrode must also not react with the solid conductor. As such, several commonly used ion blocking electrodes were tested against $MgSc_2Se_4$ (such as Ag, In, C, W), and the impedance were observed to increase over time (see evolution of Ag 1st impedance to Ag 2nd impedance in **Supplementary Figure 9**), suggesting that none of these electrodes is inert with $MgSc_2Se_4$. Inert metals, such as Au

and Pt, can be alternately used for such measurements. However, the deposition of Au and Pt onto the $MgSc_2Se_4$ pellet involves a short period of air exposure, which we found to dramatically increase the impedance (**Supplementary Figure 9**) probably through the formation of an O-containing interface layer via a reaction of $MgSc_2Se_4$ with constituents in air. One electrode which satisfies the criteria of a good ion blocking electrode is Ta, and was therefore used in our study.

The impedance data of the Ta/$MgSc_2Se_4$/Ta cell was analyzed through the generalized circuit model developed by Jamnik and Maier.[2,3] In this model, the electron and the ionic rails, which represent the electronic and ionic transport, were connected to "chemical capacitors" (**Supplementary Figure 10**). These chemical capacitors describe the capability of the bulk material to store chemical energy due to stoichiometry variations. A large chemical capacitance indicates that the material is capable to accommodate a large degree of stoichiometry change. Thus, the equivalent circuit used to fit our data is shown in **Supplementary Figure 10**. In the equivalent circuit of **Supplementary Figure 10**, the electronic rail groups the interface electron transfer resistance and capacitance ($R_e^{interface}$, $C_e^{interface}$) across the $MgSc_2Se_4$/Ta interfaces, as well as the bulk and grain-boundary electron transport resistance (R_e^{bulk}, R_e^{GB}). Similar components corresponding to the ionic transport were included in the ionic rail (i.e., $R_i^{interface}$, $C_i^{interface}$, R_i^{bulk} and R_i^{GB}) indicated in **Supplementary Figure 10**. In contrast to the original Jamnik-Maier model accounting only for the bulk component, we observed the importance of introducing another circuit element (*i.e.*, the GB element highlighted in pink, **Supplementary Figure 10**) in the fitting, since the simulated data with only one Jamnik-Maier element substantially deviates from our observation (**Supplementary Figure 11** and **Supplementary Figure 12**).

In our fitting, assuming Ta acts as an ideal ion blocking electrode, the interface ion transfer resistance was fixed to an arbitrarily large value (10^{20} Ω), and the $R_i^{interface}$ and $C_i^{interface}$ were fixed to zero. All the other circuit elements could vary during the fitting. The fitted values of these elements are Re = 4.7 M ohm, Ri = 1454 ohm, C_{chem}^{Bulk} = 220 pF, C_{chem}^{GB} = 2300 pF, $C^{interface}$ = 60 pF. Only the summed resistances from the bulk and grain boundary contributions are reported (i.e., R_e and R_i), since we believe the individual contribution of

bulk and grain boundary contributions cannot be reliably extracted due to their large degree of correlations in the current model. Mg Conductivity as function of temperature was thus measured by utilizing a $Ta/MgSc_2Se_4/Ta$ symmetric cell, from which the activation barrier results approximately 200 ± 40 meV (**Supplementary Figure 14**).
Supplementary Figure 13 shows the simulated impedance spectra utilizing different ionic resistance R_i at fixed ionic resistance R_e, refer to circuit model in **Supplementary Figure 10**.

Supplementary Note 4: NMR Spectroscopy
NMR spectroscopy is a powerful tool to study solid ion conductors and battery materials.[6–13] Therefore, ^{25}Mg solid state NMR spectroscopy has been utilized to directly probe the Mg local order and ion dynamics in $MgSc_2Se_4$. The only resonance(s) observed at 53ppm in **Supplementary Figure 15** indicate the Mg_{tet} order which has undergone motional linewidth narrowing at the NMR measurement conditions (several μs to ms) and temperatures (>240K). The narrow and Lorentzian ^{25}Mg NMR lineshape coupled with short spin lattice relaxation times indicate fast Mg dynamics. At low temperatures, a slight Gaussian broadening to the main peak has been observed which could be indicative of arrested Mg mobility or lattice defects. An application of Waugh and Fedin formula ($E_a = 1.617 \times 10^{-3}.\ T_c$, where T_c denotes the onset temperature of motional narrowing) for ion hopping activation energy,[6] based on the onset of lineshape broadening (at ~240K), suggests that the energy of activation is lower than 400 meV. This was found to be consistent with the energy of activation derived from the relaxometry analysis described in the main text.

Temperature dependent spin lattice relaxation (SLR) times for the ^{25}Mg NMR signal were derived via the logarithmic fitting of the signal intensity as shown in the representative plots in **Supplementary Figure 16**. The deviation of the experimental data from the fit is used for calculation of error bars in the main text Figure 3b. A typical relaxometry analysis based on literature study on[6,7] Li NMR of battery materials[6–13] adapted for ^{25}Mg NMR signal. Particularly, the SLR analysis and Arrhenius fitting where the details can be found in Ref. 6 for $Li_7La_3Zr_2O_{12}$ type highly lithium ion conducting garnets is closely followed

to derive similar experimental activation energies of Mg-ion hopping. The ^{25}Mg SLR NMR rates are measured at $\omega_0/2\pi$ = 18.37 MHz and are plotted in the Arrhenius diagram of main text **Figure 3b**. The mean of the Mg jump rates at the observed local maximum of 450K (**of Figure 3b**) turn out to be $1.15\text{x}10^8$ Hz. The jump rate is converted into the self-diffusion coefficient via the Einstein-Smoluchowski equation $D_{sd} = a^2/(6\tau)$ where a is the jump distance of 4.815 Å Mg_{tet} –Mg_{tet} distance in the lattice, D_{sd} turn out to be $4.53\text{x}10^{-8}$ cm^2s^{-1} at 450 K. In general, if the SLR is solely induced by diffusion processes, such as in a solid ion conductor and in the absence of paramagnetic centers, the diffusion-induced relaxation rate first increases with increasing T (low temperature range), passes through a maximum and then decreases. The SLR has been shown to be proportional to $\exp[E_a/(k_BT)]$ in the low temperature range. Assuming the maximum is at 450K, E_a from an Arrhenius fit of the plot in the main text (**Figure 3b**) turn out to be 370 ± 90 meV, consistent with both theory and motional linewidth narrowing analysis (see text above and **Supplementary Figure 15**).

Supplementary Note 5: Band Gap

The chalcogenide materials investigated show a direct band gap (Γ- Γ transition) using both the GGA-PBE and HSE06 exchange and correlation functional. The magnitude of the band gap (**Supplementary Figure 17**) decreases when moving down the chalcogenide group, i.e. $S^{2-} > Se^{2-} > Te^{2-}$.

Supplementary Note 6: Electrochemical stability of $MgSc_2Se_4$ against anode and cathode electrodes

To study the effect of electrolyte composition we analyze the stable phases of the Mg-Sc-Se chemical space using the DFT energies computed for the elements (Mg, Sc and Se), binaries and ternary compounds of **Supplementary Figure 3**. By varying the Mg chemical potential from very reducing conditions, corresponding to Mg metal anode (μ_{Mg} = 0 eV or equivalently V= 0 V) up to very oxidizing conditions (μ_{Mg} << 0 eV, V ≈ $\mu_{Mg}/2$), matching the chemical potential of Mg in the cathode (or anode), we can assess the electrochemical compatibility of the solid electrolyte against the cathode and anode electrodes. **Supplementary Figure 18** shows the grand-potential phase diagram Mg-Sc-Se at various

cathode voltages, i.e. 0 (red line, Mg anode), 0.25, 0.5, 0.75, 1.0 V and 1.25 V (blue lines), respectively. The red-dots and blue-squares indicated only the stable phases.

As the voltage is progressively decreased from 1.25 to 0 V, the $MgSc_2Se_4$ phase appears as stable above ~0.25 V, **Supplementary Figure 17**. At 0 V (the voltage of Mg metal) the $MgSc_2Se_4$ conductor is unstable, whereas $MgSc_2Se_4$ is compatible with both spinel-$MgTi_2S_4$ and Chevrel-Mo_6S_8 phase operating at ~1.2 and ~1.1 V, respectively.[14,15]

Supplementary Note 7: Mg migration in interfacial phases

As examined from the grand-potential phase diagram of **Supplementary Figure 18**, the solid ionic conductor $MgSc_2Se_4$ will decompose when in contact with Mg metal (at a 0 Volt) forming MgSe and ScSe (as from the phase diagram in **Supplementary Figure 5**). Thus, Mg ions will "percolate" through a MgSe, and the migration barriers responsible for this process are plotted in **Supplementary Figure 19**.

In **Supplementary Figure 19**, the migration barriers of Mg ions in the MgZ_2 (with Z =O, S and Se) decrease progressively along the chalcogenide group from ~ 1840 meV in MgO to ~695 meV in MgSe. These values indicate that to observe significant Mg transport at the interface of Mg metal and $MgSc_2Se_4$, the MgSe layer must be thin enough.

Supplementary Note 8: Electrochemical measurements and demonstration of Mg transport in $MgSc_2Se_4$

The electrochemical stability window of $MgSc_2Se_4$ was studied in the Ta/$MgSc_2Se_4$/Ta configuration using linear sweep voltammetry (LSV) measurements, as reported in **Supplementary Figure 20**. No current associated with $MgSc_2Se_4$ bulk decomposition was observed, consistent with XRD analysis of $MgSc_2Se_4$ after the LSV scans. However, we cannot rule out the possibility of surface decomposition which is often overlooked in LSV measurements. The Mg/$MgSc_2Se_4$/Ta configuration was not chosen, because of the likely decomposition product MgSe when $MgSc_2Se_4$ is in contact with Mg, which can block Mg diffusion based on our theoretical calculations.

Supplementary Note 9: Mg conductors screening

The class of Mg ion conductors MgX_2Z_4 was identified by the following criteria:

1) As suggested by Rong *et al.*,[4] we searched for compounds where the stable Mg site

shows an *unfavorable* coordination of ~4. For example, most Mg-containing spinels host the Mg^{2+} ions in tetrahedral coordination.

2) Simultaneously, we searched for Mg-compounds with a large volume per anion and large anion electric polarizability. A previous study[16] (see Figure S11 in the supplementary materials of Ref. 16) estimated that structures with volumes per anion larger than ~45 $Å^3$ are sufficient to achieve low activation barriers (< 600 meV) for Mg migration in a FCC-anion framework.

3) We avoided compounds containing metals that can be redox-active, i.e., metals with multiple possible oxidation states. Examples of redox-active metals include Ti, V, Cr, Mn, Fe, Co, Ni, Cu, Nb, Mo, Tc, Ru, Rh, Pd, Ag, Ta, W, Re, Os, Ir, Pt and Au. As indicated below, we also excluded compounds containing ions that can migrate together with Mg, such as H, Li, Na and K. Finally, we excluded compounds containing O to avoid poly-anion groups such as SO_4 or SeO_4, which are not representative of anions with large electric polarizability.

Following these guidelines, we screened the Materials Project database (https://www.materialsproject.org) for sulfide and selenide chemistries utilizing the query of **Supplementary Table 5**. Subsequently, we analysed the Mg coordination environment in the resultant queried structures and only chose frameworks with a tetrahedral Mg, which is indicated in **Supplementary Table 6** and **Supplementary Table 7**.

The highlighted entries (in grey) in **Supplementary Table 6** and **Supplementary Table 7** are the compounds identified by the screening criteria described above. All the highlighted compounds are in the spinel configuration MgX_2Z_4 (with X a closed shell metal and Z= S or Se), with Mg occupying corner-sharing tetrahedral sites.
Among the sulfides, we identified $MgSc_2S_4$, $MgIn_2S_4$, $MgLu_2S_4$ and $MgYb_2S_4$ as candidate frameworks, while $MgSc_2Se_4$, $MgLu_2Se_4$, $MgTm_2Se_4$, MgY_2Se_4 and $MgYb_2Se_4$, were identified among selenides. Activation barriers for Mg migration in MgX_2Z_4 with X = Lu,

Tm and Yb were not computed since these are rare-earth elements, which are known to be expensive and thereby unsuitable for large scale energy storage applications.

SUPPLEMENTARY REFERENCES

1. Jamnik, J., Maier, J. & Pejovnik, S. Interfaces in solid ionic conductors: Equilibrium and small signal picture. *Solid State Ion.* **75,** 51–58 (1995).
2. Jamnik, J. & Maier, J. Treatment of the Impedance of Mixed Conductors Equivalent Circuit Model and Explicit Approximate Solutions. *J. Electrochem. Soc.* **146,** 4183–4188 (1999).
3. Jamnik, J. & Maier, J. Generalised equivalent circuits for mass and charge transport: chemical capacitance and its implications. *Phys. Chem. Chem. Phys.* **3,** 1668–1678 (2001).
4. Rong, Z. *et al.* Materials Design Rules for Multivalent Ion Mobility in Intercalation Structures. *Chem. Mater.* **27,** 6016–6021 (2015).
5. Liu, M. *et al.* Spinel compounds as multivalent battery cathodes: a systematic evaluation based on ab initio calculations. *Energy Environ. Sci.* **8,** 964–974 (2015).
6. Kuhn, A. *et al.* Li self-diffusion in garnet-type $Li_7La_3Zr_2$ as probed directly by diffusion-induced ^{7}Li spin-lattice relaxation NMR spectroscopy. *Phys. Rev. B* **83,** 94302 (2011).
7. Kuhn, A., Duppel, V. & Lotsch, B. V. Tetragonal $Li_{10}GeP_2S_{12}$ and Li_7GePS_8 – exploring the Li ion dynamics in LGPS Li electrolytes. *Energy Environ. Sci.* **6,** 3548 (2013).
8. Kuhn, A. *et al.* NMR relaxometry as a versatile tool to study Li ion dynamics in potential battery materials. *Solid State Nucl. Magn. Reson.* **42,** 2–8 (2012).
9. Indris, S., Heitjans, P., Uecker, R. & Roling, B. Li Ion Dynamics in a $LiAlO_2$ Single Crystal Studied by ^{7}Li NMR Spectroscopy and Conductivity Measurements. *J. Phys. Chem. C* **116,** 14243–14247 (2012).
10. Heitjans, P., Schirmer, A. & Indris, S. in *Diffusion in Condensed Matter* 367–415 (Springer-Verlag, 2005).
11. Heitjans, P. & Indris, S. Diffusion and ionic conduction in nanocrystalline ceramics. *J. Phys. Condens. Matter* **15,** R1257–R1289 (2003).
12. Epp, V., Gün, Ö., Deiseroth, H.-J. & Wilkening, M. Highly Mobile Ions: Low-Temperature NMR Directly Probes Extremely Fast Li^+ Hopping in Argyrodite-Type Li_6PS_5Br. *J. Phys. Chem. Lett.* **4,** 2118–2123 (2013).
13. Epp, V., Gün, O., Deiseroth, H.-J. & Wilkening, M. Long-range Li^+ dynamics in the lithium argyrodite Li_7PSe_6 as probed by rotating-frame spin-lattice relaxation NMR. *Phys. Chem. Chem. Phys.* **15,** 7123–32 (2013).
14. Sun, X. *et al.* A high capacity thiospinel cathode for Mg batteries. *Energy Environ. Sci.* **9,** 2273–2277 (2016).
15. Aurbach, D. *et al.* Prototype systems for rechargeable magnesium batteries. *Nature* **407,** 724–727 (2000).
16. Wang, Y. *et al.* Design principles for solid-state lithium superionic conductors. *Nat. Mater.* **14,** 1026–1031 (2015).
